# Effects of Soluble Corn Fiber Alone or in Synbiotic Combination with *Lactobacillus rhamnosus* GG and the Pilus-Deficient Derivative GG-PB12 on Fecal Microbiota, Metabolism, and Markers of Immune Function: A Randomized, Double-Blind, Placebo-Controlled, Crossover Study in Healthy Elderly (Saimes Study)

**DOI:** 10.3389/fimmu.2017.01443

**Published:** 2017-12-12

**Authors:** Adele Costabile, Triana Bergillos-Meca, Pia Rasinkangas, Katri Korpela, Willem M. de Vos, Glenn R. Gibson

**Affiliations:** ^1^Health Sciences Research Centre, Life Sciences Department, Whitelands College, University of Roehampton, London, United Kingdom; ^2^Department of Food and Nutritional Sciences, University of Reading, Reading, United Kingdom; ^3^Department of Veterinary Biosciences, University of Helsinki, Helsinki, Finland; ^4^Immunobiology Research Program, Department of Bacteriology and Immunology, University of Helsinki, Helsinki, Finland; ^5^Laboratory of Microbiology, Wageningen University, Wageningen, Netherlands

**Keywords:** *Lactobacillus rhamnosus* GG, *Lactobacillus rhamnosus* GG-PB12, pilus-deficient, Soluble Corn Fiber, fecal microbiota, metabolism, immunity

## Abstract

**Background:**

The aging process leads to a potential decline in immune function and adversely affects the gut microbiota. To date, many *in vitro* and *in vivo* studies focused on the application of synbiotics (prebiotics combined with probiotics) as a promising dietary approach to affect gut microbiota composition and improved functioning of the immune system. However, studies using synbiotic preparations often have the limitation that it remains unclear whether any effect observed is a result of the prebiotic or probiotic or a synergistic effect of the combined supplement.

**Objectives:**

We investigated the effects of a probiotic *Lactobacillus rhamnosus* GG and pilus-deficient *L. rhamnosus* GG-PB12 combined with Promitor™ Soluble Corn Fiber (SCF, a candidate prebiotic) on fecal microbiota, metabolism, immunity, and blood lipids in healthy elderly persons. A prospective, double-blind, placebo controlled, randomized, single-centered, crossover study in 40 healthy elderly subjects (aged 60–80 years) was carried out. Volunteers were randomized to consume either probiotic and prebiotic as synbiotic, prebiotic or placebo (maltodextrin) during 3 weeks. Three-week washout periods separated all the treatments. We assessed effects upon blood lipids, glucose, cytokines, natural killer (NK) cell activity, phenotype, and intestinal microbiota composition. SCF decreased IL-6, which was not observed with the synbiotics.

**Results:**

Consumption of *L. rhamnosus* GG combined with SCF increased NK cell activity compared to baseline in females and the older group. In the fecal microbiota analyses, the strongest community shifts were due to *L. rhamnosus* GG combined with SCF and SCF treatments. *L. rhamnosus* GG combined with SCF and *L. rhamnosus* GG-PB12 combined with SCF significantly increased the genus *Parabacteroides*. *L. rhamnosus* GG combined with SCF and SCF increased concentrations of *Ruminococcaceae Incertae Sedis*. *Oscillospira* and *Desulfovibrio* slightly decreased in the *L. rhamnosus* GG combined with SCF group, whereas *Desulfovibrio* decreased also in the *L. rhamnosus* GG-PB12 combined with SCF group. *L. rhamnosus* GG combined with SCF reduced total cholesterol and LDL-cholesterol in volunteers with initially elevated concentrations. C-reactive protein significantly decreased during *L. rhamnosus* GG-PB12 combined with SCF intervention compared to baseline.

**Conclusion:**

In conclusion, the synbiotic combination of *L. rhamnosus* GG with SCF showed a tendency to promote innate immunity by increasing NK cell activity in elderly women and in 70 to 80-year-old volunteers and decreased TC and LDL-c in hypercholesterolemic patients. In addition, *L. rhamnosus* GG-PB12 combined with SCF demonstrated an increase in NK cell activity compared to SCF alone in older volunteers. We also found significant positive effects on the immune response, evidenced by a decrease of the pro-inflammatory cytokine IL-6. Therefore, dietary intervention with *L. rhamnosus* GG combined with SCF could be of importance in elderly as an attractive option for enhancement of both the microbial and immune systems.

## Introduction

The human gastrointestinal (GI) tract supports a rich and complex microbiota, whose composition and activities play important roles in nutrition, immunology, and certain disease processes. The composition of this microbial community is host specific, evolving throughout an individual’s lifetime and is susceptible to both exogenous and endogenous modifications. Recent interest in the function of this “organ” has illuminated a central position in health and disease. However, mechanisms by which the microbiota exerts its beneficial or detrimental influences remain largely undefined; thus, research in this area is key to future recommendations. A progressive increase in the proportion of elderly persons has led to heightened attention to their physiological needs. It is well known that immune function becomes compromised with age, an effect known as immune-senescence (1), which compromises the ability to respond to infections and develop increased protection after vaccination (2, 3). It may therefore contribute toward a higher mortality rate in older people and it has also been reported that in older people, the numbers and species diversity of putatively beneficial gut microbial groups, such as lactobacilli and bifidobacteria, as well as butyrate producers are reduced (4, 5). Appropriate dietary intervention is an attractive, safe, and non-invasive way to impact on gut bacteria and subsequent functioning of the immune system and has been proposed as an avenue to maintain microbiome homeostasis during aging (6). The use of probiotics, as live microbial food additions, is one such route (7). Experimental and clinical studies have suggested that probiotic supplementation may have beneficial effects on serum lipid profiles. Human studies have reported that certain probiotic strains boost natural killer (NK) cell (8) and phagocytic activities (8, 9) in healthy volunteers and may exert beneficial effects in inflammatory and atopic diseases, possibly by modulating pro- and anti-inflammatory cytokines (10). However, there are few studies examining the effect of probiotics on the immune function of elderly persons. Evidence exists on immune-stimulating effects of certain probiotics and the potential to use prebiotics to increase the levels of such bacteria, the latter being a selectively metabolized microbial substrate. Furthermore, by providing a probiotic at the same time as a prebiotic, conditions for survival of the live addition should be enhanced. Promitor™ Soluble Corn Fiber (SCF) is a well-known maize-derived source of dietary fiber with potential selective fermentation properties (11–14). Previous *in vivo* study has demonstrated the optimum prebiotic dose response of SCF (15). Previous *in vitro* work led to the selection of a probiotic bacterium, *Lactobacillus rhamnosus* GG, which combined with SCF, showed anti-pathogenic effects against *E. coli* and *Campylobacter* spp. (Costabile et al., unpublished data). Because of their strong mucus-binding properties, pili are thought to be major factors for binding and persistence of *L. rhamnosus* GG in the GI tract. Our purpose was to investigate the effects of *L. rhamnosus* GG combined with SCF (i.e., synbiotics) and SCF alone, on the fecal microbiota, metabolism, immunity, and blood lipids in healthy elderly subjects. Moreover, to address the essential role of the pili of *L. rhamnosus* GG, the pilus-less *L. rhamnosus* GG derivative LGG-PB12 was included into the study.

## Materials and Methods

### Intervention Study and Subjects

**Figure 1:**
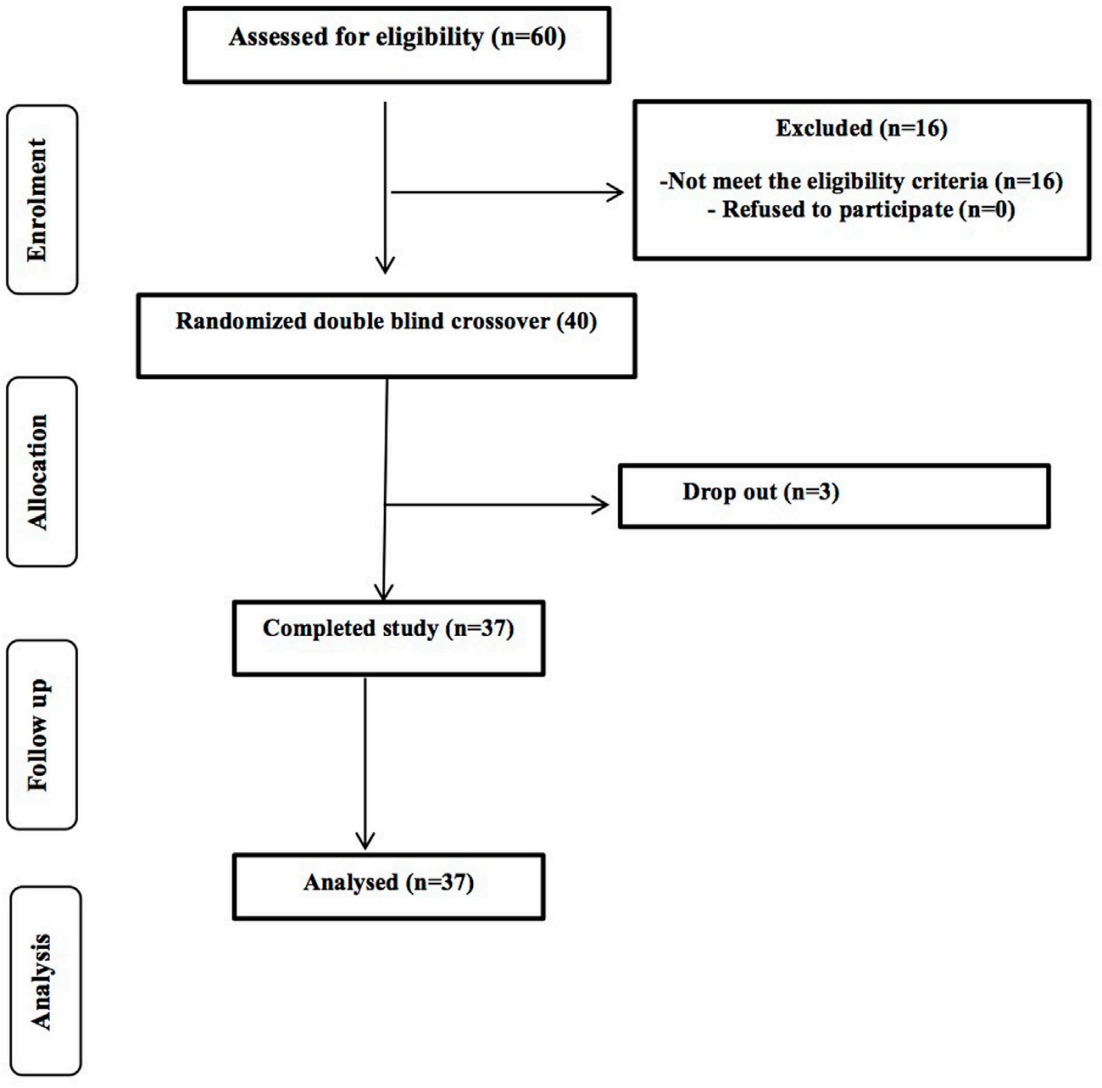
Flow of the study participants through the intervention.

A total of 80 potential healthy volunteers (aged 60–80 years) were contacted from the University of Reading and surrounding area through the Hugh Sinclair Unit of Human Nutrition volunteer database, and through advertisements within the local community between September 2014 and January 2015. Of the first 75 responded, 60 were screened and we were recruited 40 volunteers; 37 participants completed the study with 3 drop-out due to personal circumstances (Figure 1). The study was powered to get 80% statistical power (Hedwig Harvard Software) on the basis of the data from previous intervention studies in human volunteers conducted on bacteriology. We obtained written consent from each person and selection took place following determinations of health status through a medical interview and adherence to the inclusion/exclusion criteria. Inclusion criteria were as follows: aged 60–80 years, good general health, and willing to participate in the entire study. We excluded volunteers with evidence of physical or mental disease, history of drug abuse, severe allergy, or a history of severe abnormal drug reaction and smokers. Intake of an experimental drug within 4 weeks before study, former participation in a prebiotic, probiotic, or laxative trial within 2 weeks, or use of antibiotics within 6 months before the study, chronic constipation, diarrhea or other chronic GI complaint (for example, irritable bowel syndrome), lactose intolerance, and gluten allergy were all exclusion criteria. Individuals with a clinically important renal, hepatic, endocrine (including diabetes mellitus), pulmonary, pancreatic, neurologic, dermatological, urogenital/rectal, dermatological, or lymphatic disorder and any major cardiovascular condition were also excluded, as were those with a recent history, or the presence, of cancer. Any intake of drugs active on GI motility, antibiotic treatment, or any class of laxative was not permitted. Volunteers recorded any medication taken throughout the duration of the study in diaries. We instructed volunteers not to alter their usual diet or fluid intake during the trial periods; however, they were asked to refrain from consuming prebiotics and probiotics, including daily fiber intake, live yogurts, and fermented milk drinks. All volunteers signed an informed consent form, and agreed to conform to the trial guidelines and provide notification of any non-compliance.

### Study Design and Treatments

**Figure 2:**
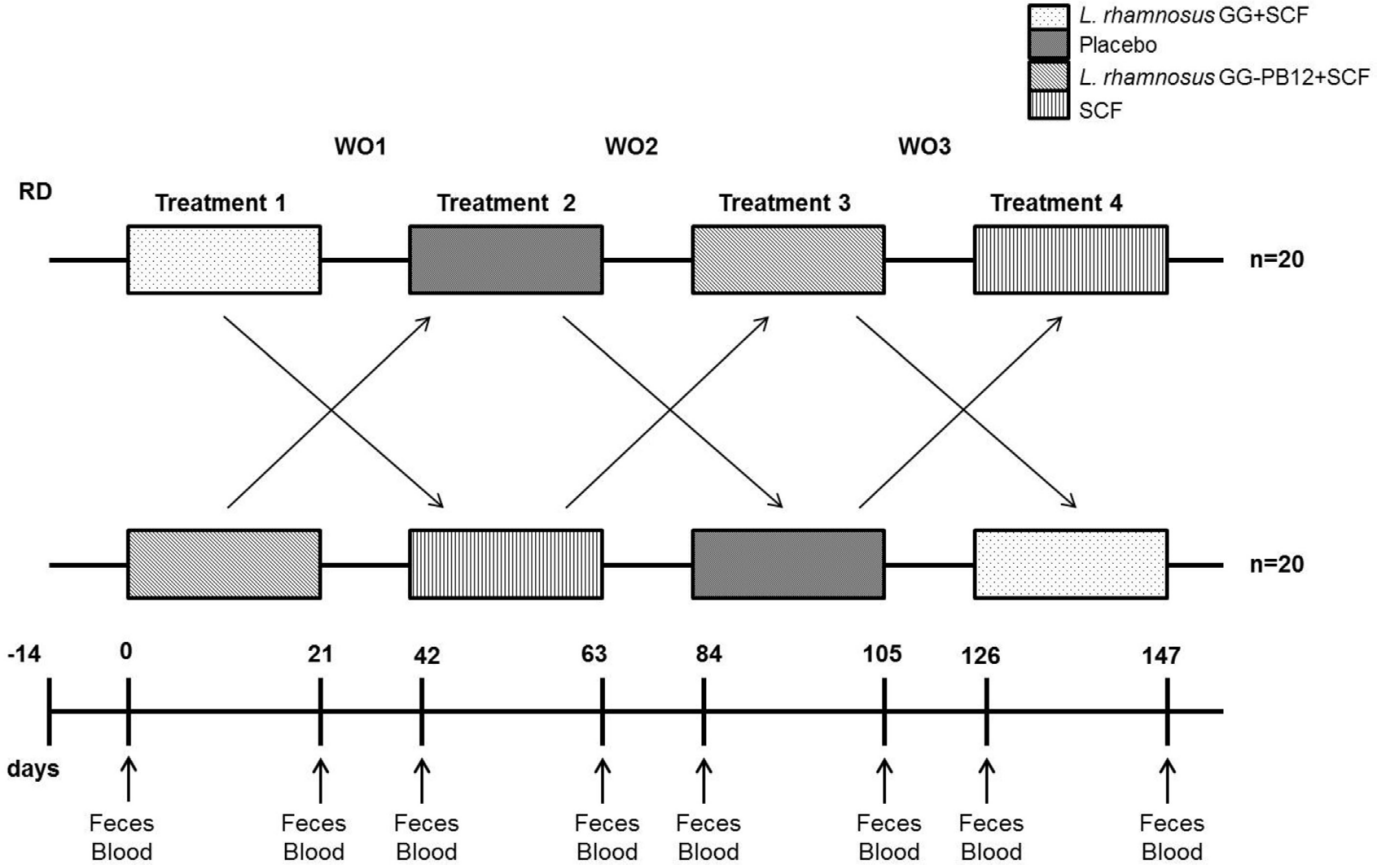
Saimes study design. The study was designed as a prospective, double-blind, randomized, placebo-controlled, single-centered crossover study with a 14 days run-in period prior to the beginning of the study, when volunteers were required to refrain from any probiotic or prebiotic containing food or drinks and control the fiber levels. The microbiota was analyzed from fecal samples taken prior to the intervention (time point 0) and at the end of the 3-week interventions (time points 21, 63, 105, and 147).

The study was registered as a clinical trial (http://clinicaltrials.gov ID: NCT03168503) and was conducted according to the Declaration of Helsinki following Good Clinical Practice (GCP). The study was approved by the University of Reading Research Ethics Committee (Ethics reference number UREC14/06). The study was designed as a prospective, double-blind, randomized, placebo-controlled, single-centered crossover study with a 2-week run-in period prior to the beginning of the study, when volunteers were required to refrain from any probiotic or prebiotic containing food or drinks and record a 4-day food diary, including one weekend day, to assess habitual diet (Figure 2). An Excel-based covariate adaptive randomization program (16) to enter four intervention arms randomized the volunteers: *L. rhamnosus* GG-PB12 combined with SCF, *L. rhamnosus* GG combined with SCF, SCF alone, or placebo (maltodextrin) stratified by age, gender, and BMI. The SCF treatment was designed and confirmed by previous study to provide a total of 6 g from SCF (Promitor™ SCF) (equivalent to 8 g/fiber delivered) (15) and was provided by Tate & Lyle, France, as dry powders and were consumed as 250 mL beverages once daily. The probiotics strains were obtained from Valio Ltd and at the dose of 1 × 10^10^ CF g. The subjects consumed one sachet (12 g) every day during 3 weeks for each treatment, with 3-week washout periods between treatments. We collected overnight 10 h-fasting venous blood and one fecal sample in the morning at baseline and at the end of each intervention period for analyses. Fecal samples were collected weekly in fecal collection kit (FC 2040, Laboratories Ltd., UK) and volunteers were asked to keep them at −80°C until the visit day when they were transferred to the laboratory for all the analysis. Throughout the pre-treatment and treatment periods, volunteers recorded details of bowel habits including stool frequency and consistency (Bristol stool scale), stomach or intestinal bloating, abdominal pain, and incidence and frequency of flatulence and stomach noises. They also completed four-day diet records in the week before baseline and each end-of-treatment clinic visit. These were analyzed using the Food Processor SQL Nutrition Analysis and Fitness 8.6.0 software (ESHA Research, Salem, OR, USA). They completed a GI symptom survey at baseline and at the end of each treatment period.

### Preparation of Peripheral Blood Mononuclear Cells (PBMC)

Fasted blood samples (10 h-fasting) were taken from volunteers in sodium heparin vacutainer tubes (Greiner Bio-One Ltd., Gloucestershire, UK). Blood was layered over an equal volume of lympholyte (Cedarlane Laboratories Limited, Tyne & Wear, UK) and centrifuged at 930 × *g* for 15 min at room temperature. Plasma was removed into a sterile tube and stored at −80°C until further use. Cells were harvested from the interface, washed once, resuspended in RPMI 1640 medium (Autogen Bioclear, Wiltshire, UK) containing 0.75 mM glutamine (Autogen Bioclear, Wiltshire, UK), and the above steps were then repeated to achieve a lower degree of erythrocyte contamination. The pellet was finally resuspended in the same medium.

### Preparation of Other Samples

Blood was collected by serum separator vacutainer tube (BD Biosciences, Oxford, UK) and centrifuged at 2,095 × *g* for 10 min. Aliquots of serum were stored at −20°C for later analysis of total cholesterol, HDL-cholesterol, triacylglycerol, non-esterified fatty acids (NEFA), glucose, and C-reactive protein (CRP).

### Blood Lipids, Glucose, and Immune/Inflammatory Markers

Concentrations of serum total cholesterol, HDL-cholesterol, triacylglycerol, NEFA, glucose, and CRP were evaluated by iLab 600 (all kits and equipment from Instrumentation Laboratory, Warrington, UK).

### Cytokine Analysis

We measured the production of IL-1β, IL-6, IL-8, IL-10, IL-12p70, and TNF using Cytometric Bead Array (CBA) multiplex kits (BD Biosciences, Oxford, UK) by flow cytometry according to the manufacturer’s instructions. BD™ CBA analysis software (BD Biosciences, Oxford, UK) was used to perform data analysis.

### NK Cell Activity

Freshly prepared PBMC were adjusted to a concentration of 5 × 10^6^ cells/mL. Viable target cells (K562 cell line) were enumerated by microscopy of trypan blue-stained cell preparations, and 5 × 10^6^ cells collected and washed twice with PBS before incubation with carboxyfluorescein diacetate succinimidyl ester (CFDA-SE) (1 µg/mL) (Sigma, Dorset, UK) for 45 min at 37°C in an air/CO_2_ (19:1) atmosphere. Following incubation, target cells were washed twice and resuspended in 1 mL of complete medium composed of RPMI 1640 medium, 0.75 mM glutamine, and 10% (v/v) newborn calf serum (Sigma, Dorset, UK). PBMC were incubated with CFDA-SE-labeled target cells for 2 h at 37°C in an air/CO_2_ (19:1) atmosphere, at effector to target cell ratios of 100:1, 50:1, 25:1, and 12.5:1. Twenty microliters of PI at 1 mg/mL were added to the samples prior to analysis by flow cytometry. Samples were acquired using a FACSCanto II flow cytometer (BD Biosciences, San Jose, CA, USA), and data analyzed using FACSDiva v6 software (BD Biosciences, San Jose, CA, USA). The results were expressed as percent lysis of the target cells.

### Lymphocyte/NK Cells Phenotype Analysis

Peripheral blood mononuclear cells were stained with appropriate combinations of fluorescently labeled mouse anti-human monoclonal antibodies for discrimination between different lymphocyte subsets. Monoclonal antibodies were conjugated to fluorescein isothiocyanate, phycoerythrin (PE), or (Cy-7 PE). PBMC were incubated with fluorescently labeled monoclonal antibodies (all from BD Biosciences, Oxford, UK) for 20 min at room temperature in the dark and washed by FACS Wash solution [1% (w/v) BSA, 0.1% (w/v) sodium azide in PBS] twice before fixing with FACS Fix solution [2% (w/v) paraformaldehyde, pH 7.4]. The fixed cells were analyzed by flow cytometry within 24 h. At least 50,000 lymphocytes were acquired using a FACSCanto II flow cytometer (BD Biosciences, San Jose, CA, USA), and data were analyzed using FlowJo V 10.0.8 software (BD Biosciences, San Jose, CA, USA). Isototypic controls were used to minimize electronic noise. Using side scatter and forward scatter, a gate was drawn in lymphocyte populations. Anti-CD3 and anti-CD56 mAb (present in all tubes) were used to define three cells populations: conventional T cells (CD3^+^CD56^−^), conventional NK cells (CD3^−^CD56^+^), and NKT cells (CD3^+^CD56^+^). Anti-CD56 and anti-CD16 mAb (present in all tubes) were used to further divide NK cells based in CD56 expression into two subsets: CD56^dim^ and CD56^bright^. Gates strategies were designed based on Almeida-Oliveira et al. (17). Results of phenotypes were expressed as percentage of the subsets or within the cell group specified.

### DNA Extraction and Quantitative PCR Analysis

DNA extraction from feces was carried out using the RBB method as described by Salonen et al. (18). Following cell lysis and precipitation of nucleic acids, QIAamp DNA Minikit was used for further purification of the DNA. Afterward, compliance of the study subjects to the trial was verified using quantitative PCR assays. The assays were designed to target the position of one of the mutations, 443187, in *srtC1* gene in *L. rhamnosus* GG-PB12 genome (19) (Figure S1 in Supplementary Material). Primers for both *L. rhamnosus* GG and GG-PB12 assays were the same (for 5′-AGTGCGACTATTAGCTTTA-3′ and Rev 5′-GGATCTTGTGACCTTAATG-3′), while the probes contained one nucleotide difference (*L. rhamnosus* GG: 5′-(FAM) TTGTT**C**CACCAAACC (MGB-NFQ)-3′; *L. rhamnosus* GG-PB12: 5′-(FAM) TTGTT**T**CACCAAACC (MGB-NFQ)-3′, nucleotide difference marked in bold). Probes were non-fluorescent quenched minor groove binder (NFQ-MGB) hydrolysis probes with 6-fluorescein amidite (6-FAM) as the fluorophore. Nucleotide oligo (5′-CGACTATTAGCTTTAATTTGTTC-3, T_m_ 48°C) was used in the *L. rhamnosus* GG-PB12 assay to reduce potential non-specific signal from strain GG. The oligo was designed so that it anneals to the binding site of the *L. rhamnosus* GG-specific probe before the temperature reaches the probe annealing temperature and blocks the binding site of the probe. HOT FIREPol^®^ Probe qPCR Mix Plus with ROX (Solis BioDyne Ltd.) was used in the qPCRs with program 15 min at 95°C, 20 s at 95°C, and 1 min at 60°C, for 35 cycles. 25 ng of DNA was used per reaction in a total volume of 20 µL. In addition, total bacteria were quantitated using an qPCR assay described by Nadkarni et al. (20), with total DNA quantity of 0.5 ng per reaction and using master mix HOT FIREPol^®^ EvaGreen^®^ qPCR Mix Plus (Solis BioDyne Ltd.). Measurements were performed with Stratagene MX3005P real-time PCR equipment and analysis of the data was performed using MxPro qPCR Software version 4.1 (Stratagene).

### Intestinal Microbiota Profiling Analyses

Comprehensive intestinal microbiota profiling and phylogenetic analysis was carried out with 16S rRNA amplicon sequencing by Illumina Miseq. In the paired-end mode, the variable region V3–V4 was sequenced, using primers (CTACGGGNGGCWGCAG and GACTACHVGGGTATCTAATCC). However, we only kept the forward reads, truncated to 150 bases, as we have observed, using artificial microbial communities, that longer reads result in distorted microbiota compositions. Pre-processing and statistical analyses of the reads were done using the R package mare (Korpela 2016 mare: Microbiota Analysis in R Easily. R package version 1.0). To eliminate sequencing errors, we discarded all unique reads occurring <100 times in the total dataset. Taxonomic annotation of the reads was conducted using USEARCH (Edgar 2010 Bioinformatics).

### Statistics

Data on immunology and metabolism were analyzed using SPSS version 21.0. Significant differences between baseline and end of intervention were evaluated by paired *t*-test, while significant differences between treatments were evaluated by two-way analysis of variance (ANOVA) using the general linear model with Bonferroni correction. All data are shown as mean ± SE. The statistical significance level was defined as *p* < 0.05 with *p* values represented in the figures as ***p < 0.001, **p < 0.01, and *p < 0.05. Effect of the different treatments on genus-level microbiota composition was assessed using ANOVA. The change in the relative abundance of each genus during each of the synbiotic and SCF treatments was compared to changes during the placebo treatment, keeping baseline abundance of the focal genus as a covariate in the model. *p* values were corrected for multiple testing using the false discovery rate method (21). The effect of treatments on overall microbiota was assessed using permutational multivariate ANOVA (function adonis in R-package vegan) and principal coordinates analysis, using the Bray–Curtis dissimilarities (function capscale in R-package vegan).

## Results

### Blood Lipids, Glucose, Cytokines, NK Cell Activity, Phenotype, and Intestinal Microbiota Composition

**Figure 3:**
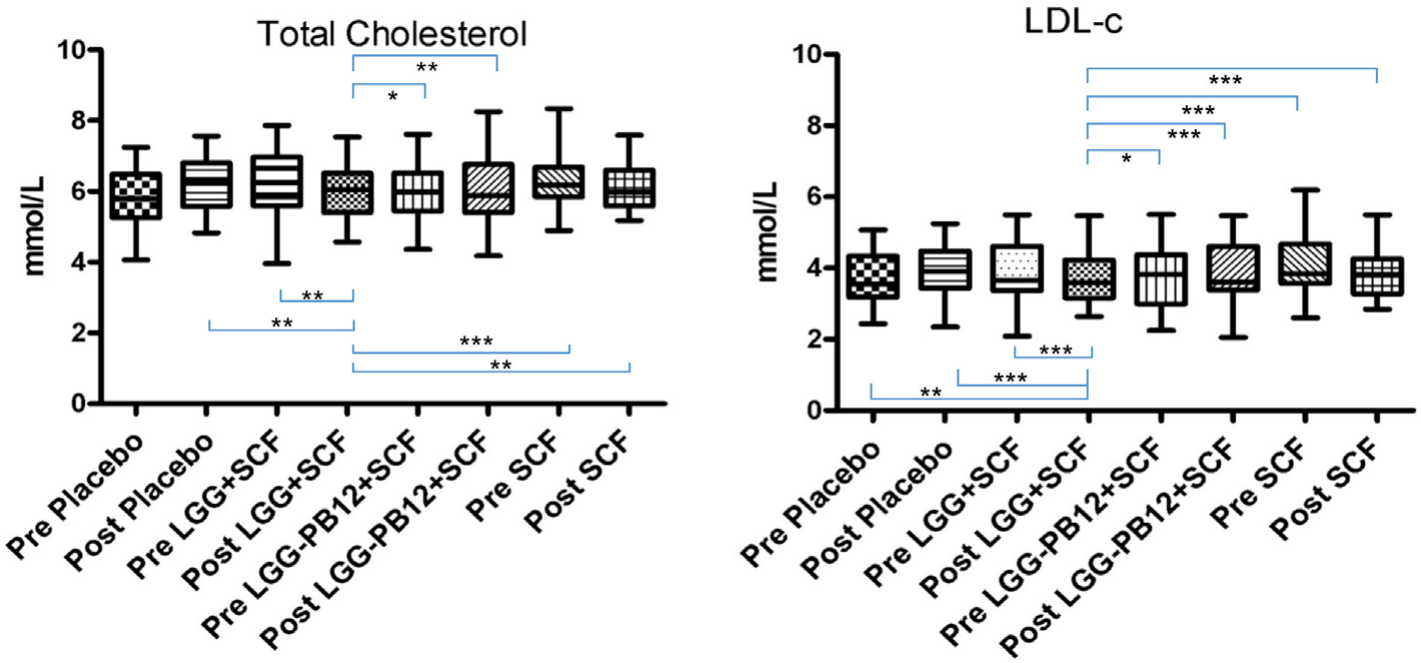
Total cholesterol **(A)** and LDL-cholesterol concentrations **(B)** in each group after the treatment, in volunteers with raised cholesterol (total cholesterol >5 mmol/L) at baseline (*n* = 26) (*p < 0.05, **p < 0.01, and ***p < 0.001).

**Figure 4:**
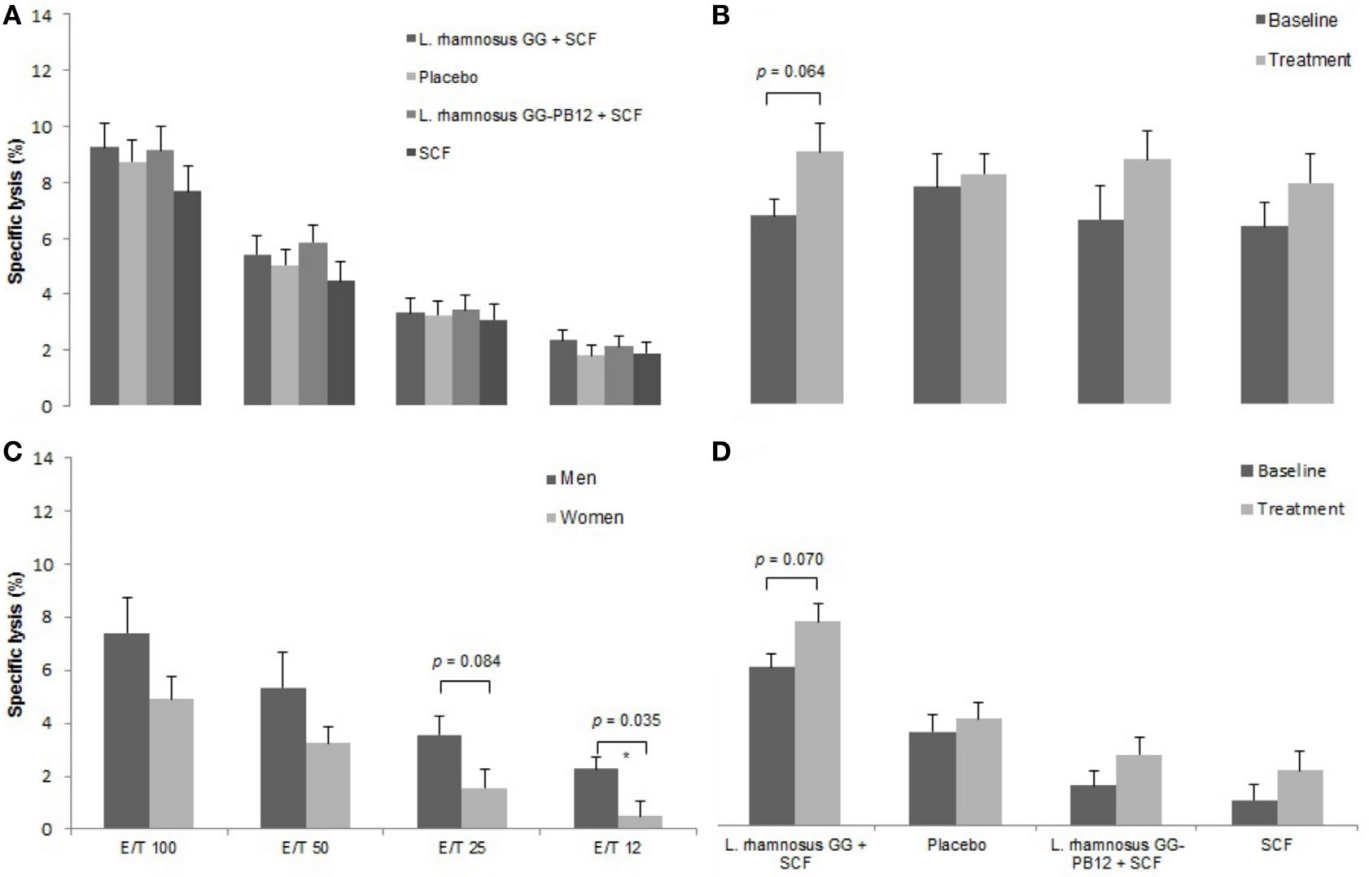
Natural killer cell activity in each group after the treatment. Data are mean ± SE. **(A)** Specific lysis after all treatments. **(B)** Specific lysis after LGG + Soluble Corn Fiber (SCF) intervention in females. *E*/*T* = 100/1. **(C)** Specific lysis at the beginning of the study in females and males. **(D)** Specific lysis after all treatments in the older group (70–80 years old) (*n* = 37).

**Figure 5:**
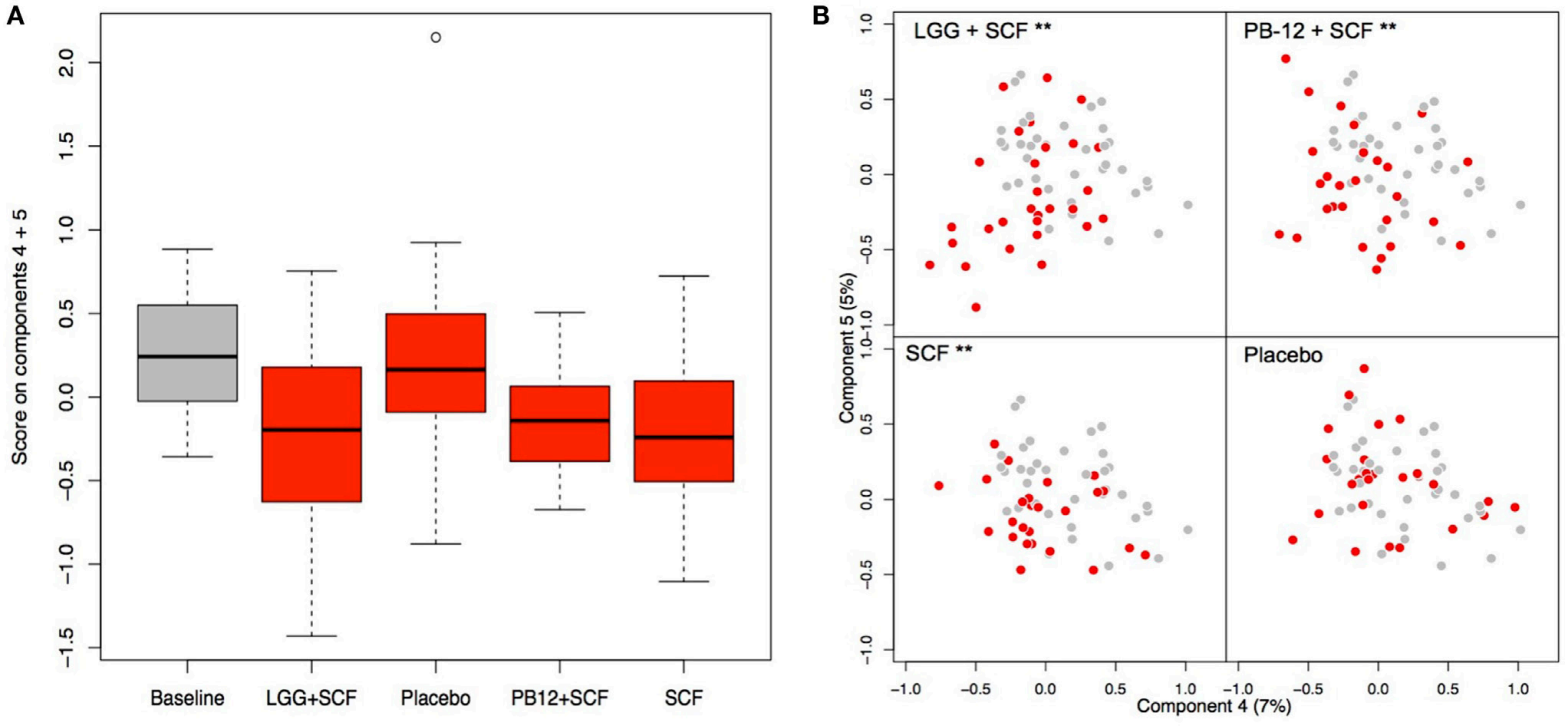
Principal coordinate analysis (PCoA) of the fecal microbiota composition in each group after the treatments. **(A)** Score on components 4 and 5 in the PCoA plots shown as a box plot; **(B)** PCoA plots indicating the shift between baseline samples (gray) and treatment samples (red) of each participant arm: LGG + Soluble Corn Fiber (SCF)**, PB12 + SCF**, SCF** (**p = 0.001), and placebo (*p* = 0.95).

A total of 37 volunteers completed the study. Intestinal symptoms, anthropometric values and blood pressure of the volunteers are shown in Tables 1–3, respectively. Overall, no statistically significant differences were found. There were no significant differences between treatments for serum TC, HDL-cholesterol, LDL-cholesterol, triacylglycerol, NEFA, or glucose (Table 4). However, after analyzing blood lipid results from volunteers with raised cholesterol (TC > 5 mmol/L) at baseline (*n* = 26), a statistically significant decrease in TC and LDL-c was observed after synbiotic intervention with *L. rhamnosus* GG combined with SCF, but not with the other treatments (Figure 3). Concentrations of IL-1β, IL-6, IL-8, IL-10, IL-12p70, TNF, and CRP were measured in serum. The cytokines IL-1β, IL-10, IL-12p70, and TNF were undetectable or only very low levels were found in the samples from elderly people (data not shown). Therefore, only IL-6, IL-8, and CRP results were analyzed (Table 5). Even though CRP serum concentrations of the subjects treated with *L. rhamnosus* GG-PB12 combined with SCF and SCF alone were higher than that with *L. rhamnosus* GG combined with SCF and placebo treatments, there were no significant differences between these. The use of SCF alone led to a decrease in the pro-inflammatory cytokine IL-6, which was not observed with the synbiotics. Although highest numbers for NK cell activity were obtained after synbiotic treatments for all ratios, no significant differences were found (Figure 4A). Therefore, there was no effect of synbiotic on NK cell activities compared to other treatments. When NK cell activity was expressed on a per cell basis (divided by NK cell percentage in lymphocytes), similar results were found (data not shown). When gender was considered, NK cell activity after *L. rhamnosus* GG combined with SCF intervention almost reached significance for the *E*/*T* of 100 (*p* = 0.064) compared to baseline in females but not in males (Figure 4B). Furthermore, NK cell activity at the beginning of the study was significantly higher in males than in females (Figure 4C). Interestingly, when dividing volunteers by age, i.e., 60–69 and 70–80 years old, NK cell activity after *L. rhamnosus* GG combined with SCF intervention almost reached significance in the latter group (Figure 4D). Finally, *L. rhamnosus* GG-PB12 combined with SCF increased NK cell activity compared to SCF alone in older volunteers, showing tendency in *E*/*T* = 100 (*p* = 0.079) and significance when expressed as log *E*/*T* = 100 (*p* = 0.042). There were no significant effects of synbiotics on lymphocyte and NK cell phenotypes (data not shown). Quantities of the *L. rhamnosus* GG strains and total bacteria, in feces, were measured using quantitative PCR (Table 6). Not surprisingly, increased numbers of *L. rhamnosus* GG or *L. rhamnosus* GG-PB12 were detected during intervention with the synbiotic in question, but not during placebo or SCF intervention. Impact of the interventions on total microbial communities was addressed by phylogenetic analysis of the fecal microbiota (Figure 5). *L. rhamnosus* GG combined with SCF, *L. rhamnosus* GG-PB12 combined with SCF, and SCF treatments had significant effects on overall microbiota composition when compared to baseline, each treatment explaining 2% of the variation in microbiota compositions (*p* = 0.001). All active treatments caused a similar microbiota shift along principal components 4 and 5, toward higher *Parabacteroides* and *Ruminococcaceae* abundance; the strongest shift was seen in *L. rhamnosus* GG combined with SCF and *L. rhamnosus* GG-PB12 combined with SCF treatments. The placebo treatment did not have a significant impact compared to baseline levels on overall microbiota composition (*R*^2^ = 0.03, *p* = 0.95) compared to the baseline. Significant changes were detected in four bacterial genera in the different treatments. *Parabacteroides* was significantly increased during both synbiotic treatments (*L. rhamnosus* GG: 4.3% increase compared to placebo, *p* = 0.0004; *L. rhamnosus* GG-PB12: 3.4% increase, *p* = 0.0075). *Ruminococcaceae* Incertae sedis was increased significantly *L. rhamnosus* GG synbiotic treatment and treatment with SCF only (*L. rhamnosus* GG: 2.4% increase, *p* = 0.014; SCF: 2.4% increase, *p* = 0.015). A subtle, but significant, decrease in *Oscillospira* was detected following *L. rhamnosus* GG combined with SCF treatment (0.04%, *p* = 0.012), while a significant decrease in *Desulfovibrio* was detected during both synbiotic treatments (*L. rhamnosus* GG: 0.09% *p* = 0.011; *L. rhamnosus* GG-PB12: 0.1%, *p* = 0.0075).

**Table 1:** Average, self-reported, gastrointestinal symptom scores for stool frequency, consistency, bloating, flatulence, and abdominal pain, over the 3-week intervention period for the placebo, synbiotics, and SCF for each treatment group (*n* = 37).

	Placebo	*Lactobacillus rhamnosus* GG + SCF	*L. rhamnosus* GG-PB12 + SCF	SCF
Stool frequency^a^	1.95 ± 1.30	1.95 ± 1.51	1.98 ± 1.65	1.99 ± 1.56
Stool consistency	3.82 ± 0.99	3.86 ± 1.06	4.15 ± 0.99	3.81 ± 1.05
Intestinal bloating^b^	0.22 ± 0.44	0.17 ± 0.39	0.14 ± 0.26	0.15 ± 0.39
Flatulence^b^	0.46 ± 0.50	0.56 ± 0.57	0.51 ± 0.62	0.53 ± 0.56
Abdominal pain^b^	0.11 ± 0.19	0.09 ± 0.21	0.15 ± 0.31	0.15 ± 0.32

*SCF, Promitor™ Soluble Corn Fiber*.

*^a^Number of stools per day. Estimated using the Bristol stool chart (scale with seven stool types; Type 1—hard to Type 7—entirely liquid)*.

*^b^Overall scale from 0 to 3 (0—no symptoms; 1—very mild symptoms to 3—very severe symptoms). All values are means ± SD. No significant differences were shown between baseline and post-intervention or between treatments and control. Two-way analysis of variance with Bonferroni correction were used to assess treatment, time, and treatment by time interaction effects*.

**Table 2:** Change in anthropometric measurements in all study participants (*n* = 37) between baseline and 3 weeks intervention period with placebo (maltodextrin), *Lactobacillus rhamnosus* GG + SCF, *L. rhamnosus* GG-PB12 + SCF, and SCF only.

	Weight (kg)		BMI (kg/m^2^)		Waist (cm)	
	Female	Male	Female	Male	Female	Male
Baseline	68.01 ± 7.42	85.18 ± 10.86	25.48 ± 3.31	27.11 ± 2.21	89.85 ± 8.65	97.78 ± 10.24
Placebo	67.71 ± 7.49	84.46 ± 11.06	25.52 ± 3.36	27.40 ± 2.41	86.40 ± 9.09	97.40 ± 12.29
*L. rhamnosus* GG + SCF	68.01 ± 7.48	85.18 ± 11.27	25.58 ± 3.55	27.55 ± 2.44	86.95 ± 9.00	99.05 ± 11.32
*L. rhamnosus* GG-PB12 + SCF	67.60 ± 6.82	83.26 ± 10.86	25.78 ± 3.31	26.97 ± 2.41	86.02 ± 9.51	97.50 ± 7.93
SCF	67.58 ± 6.70	84.98 ± 10.62	25.81 ± 3.32	27.32 ± 2.31	88.46 ± 8.25	99.40 ± 9.12

*SCF, Promitor™ Soluble Corn Fiber*.

*Values represent means ± SD. No significant difference was shown between baseline, intervention, and post-intervention. Two-way analysis of variance with Bonferroni correction were used were used to assess treatment, time, and treatment by time interaction effects*.

**Table 3:** Blood pressure in each group after the treatment (*n* = 37).

	Systolic (mm Hg)		Diastolic (mm Hg)	
	Female	Male	Female	Male
Baseline	136.62 ± 18.26	135.27 ± 11.29	73.12 ± 7.98	77.82 ± 6.59
Placebo	132.46 ± 18.11	132.20 ± 12.80	71.48 ± 8.09	75.00 ± 7.90
*L. rhamnosus* GG + SCF	135.40 ± 20.72	139.70 ± 15.27	71.96 ± 8.74	78.70 ± 5.48
*L. rhamnosus* GG-PB12 + SCF	133.92 ± 19.17	132.20 ± 9.62	70.96 ± 8.18	77.00 ± 7.79
Soluble Corn Fiber	134.42 ± 22.29	131.83 ± 12.34	70.08 ± 7.91	75.50 ± 4.66

*All values are means ± SD. No significant differences were shown between baseline and post-intervention (two-way analysis of variance with Bonferroni correction)*.

**Table 4:** Lipid parameters baseline and 3 weeks intervention study in the active [*Lactobacillus rhamnosus* GG + Soluble Corn Fiber (SCF), *L. rhamnosus* GG-PB12 + SCF, and only SCF] and placebo treatment groups (*n* = 37).

	TC (mmol/L)	Change from baseline	HDL-c (mmol/L)	Change from baseline	LDL-c (mmol/L)	Change from baseline	TG (mmol/L)	Change from baseline	Non-esterified fatty acids (mmol/L)	Change from baseline	Glucose (mmol/L)	Change from baseline	TC:HDL-c ratio	Change from baseline
Normal range	<5		>1		<3		<1.7		0.1–0.9		4–5.9		<4	
Pre-placebo	5.54 ± 0.16		1.57 ± 0.06		3.48 ± 0.14		1.07 ± 0.05		0.42 ± 0.03		5.29 ± 0.14		3.60 ± 0.11	

Post-placebo	5.79 ± 0.17	0.317 ± 0.21	1.65 ± 0.06	0.104 ± 0.06	3.61 ± 0.14	0.161 ± 0.15	1.17 ± 0.08	0.114 ± 0.07	0.41 ± 0.03	−0.006 ± 0.03	5.35 ± 0.12	0.196 ± 0.16	3.63 ± 0.13	0.090 ± 0.12

Pre-*L. rhamnosus* GG + SCF	5.72 ± 0.18		1.57 ± 0.05		3.58 ± 0.16		1.15 ± 0.07		0.47 ± 0.04		5.35 ± 0.12		3.69 ± 0.13	

Post-*L. rhamnosus* GG + SCF	5.73 ± 0.14	0.161 ± 0.22	1.67 ± 0.06	0.187 ± 0.10	3.51 ± 0.12	0.127 ± 0.17	1.19 ± 0.08	0.072 ± 0.07	0.44 ± 0.03	−0.017 ± 0.05	5.40 ± 0.13	0.203 ± 0.19	3.52 ± 0.12	0.027 ± 0.12

Pre-*L. rhamnosus* GG-PB12 + SCF	5.62 ± 0.16		1.66 ± 0.06		3.47 ± 0.15		1.09 ± 0.06		0.42 ± 0.03		5.27 ± 0.11		3.51 ± 0.14	

Post-*L. rhamnosus* GG-PB12 + SCF	5.69 ± 0.10	0.095 ± 0.12	1.62 ± 0.06	−0.026 ± 0.03	3.54 ± 0.17	0.095 ± 0.09	1.16 ± 0.19	0.059 ± 0.05	0.44 ± 0.03	0.015 ± 0.03	5.39 ± 0.06	0.140 ± 0.09	3.59 ± 0.13	0.100 ± 0.06

Pre-SCF	5.97 ± 0.18		1.70 ± 0.06		3.76 ± 0.17		1.12 ± 0.07		0.43 ± 0.04		5.42 ± 0.11		3.62 ± 0.15	

Post-SCF	5.82 ± 0.15	0.020 ± 0.18	1.64 ± 0.05	−0.005 ± 0.05	3.63 ± 0.14	−0.021 ± 0.14	1.20 ± 0.08	0.102 ± 0.06	0.44 ± 0.03	0.019 ± 0.04	5.53 ± 0.14	0.235 ± 0.17	3.65 ± 0.13	0.122 ± 0.13

*Data shown as means ± SE. No significant differences. Paired t-test*.

**Table 5:** Cytokine production and C-reactive protein (CRP) concentrations expressed in millimolar as change from baseline and 3 weeks intervention study in the active [*Lactobacillus rhamnosus* GG + SCF, *L. rhamnosus* GG-PB12 + Soluble Corn Fiber (SCF), and only SCF] and placebo treatment groups (*n* = 37).

	IL-6 (pg/mL)	Change from baseline	IL-8 (pg/mL)	Change from baseline	CRP (mg/L)	Change from baseline
Placebo	0.710 ± 0.242	−0.126 ± 0.236	3.551 ± 0.380	0.063 ± 0.329	1.200 ± 0.180	0.436 ± 0.176
*L. rhamnosus* GG + SCF	0.988 ± 0.245	0.309 ± 0.242	3.650 ± 0.383	0.359 ± 0.337	1.209 ± 0.182	−0.091 ± 0.177
*L. rhamnosus* GG-PB12 + SCF	0.878 ± 0.245	0.197 ± 0.241	3.528 ± 0.382	−0.167 ± 0.336	1.244 ± 0.189	−0.342 ± 0.179*
SCF	0.658 ± 0.258	−0.072 ± 0.257*	3.447 ± 0.394	0.450 ± 0.365	1.375 ± 0.188	0.024 ± 0.180

*Data shown as means ± SE*.

*Significantly different from placebo (*p* < 0.05). Paired *t*-test.

**Table 6:** Median quantities of total bacteria, *Lactobacillus rhamnosus* GG, and *L. rhamnosus* GG-PB12 in 23 fecal samples.

Treatment	Total^a^	LGG^b^	PB12^b^	Samples
LGG + SCF	5.881 × 1012	3.898 × 1010	N.D.	37
WO	4.653 × 1012	0.000	0.000	30
Placebo	4.757 × 10^12^	0.000	0.000	37
WO	4.887 × 10^12^	0.000	0.000	28
PB12 + SCF	5.429 × 10^12^	0.000	2.166 × 10^10^	37
WO	7.639 × 10^12^	0.000	0.000	26
SCF	5.919 × 10^12^	0.000	0.000	37
WO	6.100 × 10^12^	0.000	0.000	27

*^a^Quantities as 16S rRNA gene copies/g feces*.

*^b^Quantities as CFU/g feces*.

*Samples: quantity of samples in the treatment in question*.

*WO, washout; N.D. indicates not detected; SCF, Soluble Corn Fiber*.

## Discussion

The health-promoting properties of probiotics are suggested to be strain dependent; therefore, strain identification and characterization are essential (22). Probiotics elicit their functions in different ways, among which immunomodulation is suggested to be one mechanism (23). Current US national guidelines for CVD risk reduction are primarily focused on strategies to reduce concentrations of LDL-c, with the most recent focus being “lower is better” (23). Some components of the immune response, including phagocytosis, NK cell activity, and mucosal immunoglobulin A production, have been shown to be improved by certain probiotic bacterial strains, while other components, including lymphocyte proliferation, production of cytokines, and elevation of antibodies other than immunoglobulin A, appear to be less sensitive to probiotic modulation (24). NK cells are critical for the removal of intracellular pathogens and also possess vital tumoricidal activities, but their activity declines with aging (25), even though numbers of NK cells in the circulation increase with age (26). NK cell activity has been demonstrated to be enhanced by different probiotic strains studied in healthy adults (8, 9, 27) and in healthy older subjects (28, 29), Dong et al. (30) showed that six different probiotic strains enhanced activation of all NK cell subsets and elicited similar capacity to stimulate NK cell activities, without showing any strain-specific properties. This property of probiotic strains has been confirmed by human studies, in which oral consumption of *Lactobacillus casei* Shirota (LcS) (31), *Lactobacillus fermentum* CECT5716 (32), *Lactobacillus paracasei* NCC 2461 (33), *Bifidobacterium lactis* HN019 (34, 35), or *L. rhamnosus* HN001 (36, 37), enhanced NK cell activity in healthy adult or elderly populations. However, some human studies did not show any effect of probiotic strains (*L. rhamnosus, B. lactis*, LcS, *Lactobacillus gasseri* PA 16/8, *Bifidobacterium longum* SP 07/9, *Bifidobacterium bifidum* MF 20/5) on NK cell activity (38, 39), perhaps due to differences in sample size, probiotic dose, consumption period, study design, and volunteers. In our study, the *L. rhamnosus* GG combined with SCF intervention showed a tendency to increase NK cell activity compared to baseline in women. We found higher baseline NK activity values in males compared to females, which has been reported by other authors (7, 40), who also demonstrated that LcS consumption had a greater effect on female compared to male volunteers, and this effect might relate to an initially lower NK cell activity in females. Nagao et al. (41) have shown that daily intake of fermented milk containing LcS-enhanced NK cell activity in healthy young subjects with relatively lower NK cell activity. In the current study, after randomizing the volunteers by age, the *L. rhamnosus* GG combined with SCF intervention also showed a tendency to increase NK cell activity compared to baseline in elderly whose average age was 70–80 years. A possible lower baseline NK cell activity in this population could be related to the increase. It may then be suggested that the synbiotic does not necessarily enhance immune function above “normal” levels in healthy individuals, but rather modulates it back to normal levels in situations where immunity is impaired and thus plays a role in homeostasis, as previously reported for LcS (7). Flow cytometry analysis showed no difference in the relative proportion of NK cells and subsets in groups consuming the different treatments, suggesting that these synbiotics demonstrated a tendency to enhance NK cell activity but did not induce the expansion of NK cells. Overall, we have shown that synbiotic supplementation could slow down the reduction of NK cell activity without changing the relative proportion of NK cells, as previously found by Morimoto et al. (42) for LcS. In fact, these authors reported that NK cells increasing after supplementation of probiotics may be dependent on the species of lactic acid bacteria and/or the condition of the host (such as healthy subjects versus either cancer patients, or younger versus aged subjects, etc.). NK cell activity is important for immune surveillance against cancer and pathogenic infections (43, 44). On the other hand, environmental and psychological condition and NK cell activity are closely related (42). The reported effects of probiotics on cytokine production are complex and suggest strain-specific differences (31, 33). A range of cytokines was examined in the current study, but there were no significant changes in cytokine production by the synbiotics. Other human studies with probiotic interventions showed no changes in stimulated cytokine production (7, 45). In the present study, a significant decrease of pro-inflammatory IL-6 was only observed during the intervention with SCF and not in synbiotic combination. It is well known that appropriate dietary intervention is an important way to impact on gut bacteria and subsequent functioning of the immune system (6), and prebiotics have demonstrable benefits. Shukla et al. (46) reported that prebiotic supplementation modulated gut morphology and improved immune status in infected mice, while Marciano et al. (47) found that oligofructose decreased the concentration of IL-6 in rats. In the same way, Aliasgharzadeh et al. (48) observed that patients supplemented with resistant dextrin exhibited a significant decrease in IL-6 concentrations than those supplemented with maltodextrin. Childs et al. (49) demonstrated that XOS induced bifidogenesis, improved aspects of the plasma lipid profile, and modulated markers of immune function in healthy adults. A possible limitation associated with the study was that we did not specifically select subjects who were immunocompromised. The hypothesis of this study was that synbiotic intervention would at least partially reverse some of the decline in immune parameters caused by aging, although it cannot be ascertained whether the subjects in the current study were immunocompromised. Immune senescence is observed in healthy aging, and the purpose of this study was to investigate an older group, which was expected to have some degree of immune-senescence. It could be argued that the results from this study could also be applied to younger subjects and to those who are not necessarily immunocompromised. Finally, a limitation of all studies employing immune biomarkers in the absence of clinical outcomes is that it is difficult to interpret the biological significance of minor immunomodulatory effects. It is of specific interest to note that the reduction of these risk factors was only obtained with the synbiotic intervention *L. rhamnosus* GG combined with SCF but not the isogenic *L. rhamnosus* GG-PB12 that only differ in the production of mucus-binding pili (19). Furthermore, in regard to the fecal microbiota changes, an increased amount in the quantities of *L. rhamnosus* GG strains and *L. rhamnosus* GG-PB12 were detected during the intervention with the synbiotic in question, but not during placebo or SCF intervention. There are indications that *L. rhamnosus* GG persists longer in the intestinal tract of healthy adult volunteers than *L. rhamnosus* GG-PB12 (in preparation, Rasinkangas et al.). We could not address this in the present study as assessment of persistence requires several samplings during the washout period. The 2-week washout was sufficient to eliminate effectively either of the *L. rhamnosus* strains. Both isogenic strains showed differential effects on the intestinal microbiota analysis and significant changes were detected in four bacterial genera. *Parabacteroides* was significantly increased during both synbiotic treatments, whereas a significant decrease in *Oscillospira* was observed. We also observed a significant decrease in numbers of *Desulfovibrio* spp. for both symbiotic treatments. This group of sulfate-reducing bacteria has been implied in as potential players in the etiology of various intestinal disorders, including inflammatory bowel disease and colon cancer (50). Hence, their reduction by the synbiotic treatment with either of the *L. rhamnosus* GG strains may be a relevant example of their probiotic activity. As reported by Nakamura et al. (51), the acetogens, methanogenic archaea, and sulfate-reducing bacteria, which dispose the colonic hydrogen gas generated during fermentation, are low in abundance, but critical for functioning of the gut ecosystem. It is very likely that the organisms we found may be indicator species, particularly sensitive to the environment and therefore informative of important structural or functional differences between ecosystems, which lead to the differential responses. Not surprisingly, *Ruminococcaceae Incertae sedis* was increased significantly during *L. rhamnosus* GG combined with SCF treatment and SCF treatment alone, but the increase in SCF was more pronounced, indicating a possible role for this group as active plant degraders. The *Ruminococcaceae* are one of the most abundant families from the order *Clostridiales* found in the mammalian gut environment, and have been associated with maintenance of gut health. Abundance estimates, based on *16S* rRNA surveys, suggest that *Ruminococcaceae* are the most abundant Firmicute families in gut environments, accounting for roughly 50 and 30% of phylotypes, respectively (52, 53). One key point is that in gut environments the ability to degrade cellulose and hemicellulose components of plant material enables members of the *Ruminococcaceae* to decompose substrates that are indigestible by the host. Declines in *Ruminococcaceae* and *Parabacteroides* have been identified as main microbial shifts associated with aging in mice (54, 55). The fact that the *L. rhamnosus* GG combined with SCF treatment caused an increase specifically in these groups suggests that the treatment may have positive effects on the microbiota of elderly people. Elevated serum TC, LDL-c, and TAG concentrations and low HDL-c concentrations are well-established risk factors for CVD (56–58). Current US national guidelines for CVD risk reduction are primarily focused on strategies to reduce concentrations of LDL-c, with the most recent focus being “lower is better” (57). Based on meta-regression analysis of randomized trials of statins, Delahoy et al. (59) concluded that there was a significant positive relationship between reductions in LDL-c and reductions in the risk for major cardiovascular events. To date, experimental and clinical studies have suggested that probiotic supplementation may have beneficial effects on serum lipid profiles. However, there are conflicting results on the efficacy of probiotic preparations in reducing serum cholesterol. Recently, Shimizu et al. (60) evaluated the effects of probiotics on human serum lipid concentrations in a meta-analysis of interventional studies. Sadrzadeh-Yeganeh et al. (61) have reported that probiotic interventions (including fermented milk products and probiotics) produced changes in TC and LDL-c, while HDL-c and TAG concentrations did not differ significantly between probiotic and control groups. Moreover, they concluded that long-term (>4-week) probiotic intervention was statistically more effective in decreasing TC and LDL-c than short-term (<4-week) intervention. Interestingly, Shimizu et al. (60) found that decreases in TC and LDL-c concentrations during probiotic intervention were greater in mildly hypercholesterolemic than in normocholesterolemic individuals. These results have been confirmed in our findings, since *L. rhamnosus* GG combined with SCF decreased TC and LDL-c compared to the other treatments in volunteers with raised cholesterol at baseline. Therefore, synbiotic intervention will have more benefit in patients with hypercholesterolemia than in individuals with normal lipid concentrations. In addition, reductions in TC and LDL-c in the elderly have been reported to be greater than those in younger individuals. This could be considered to be due to differences in baseline values, as in the elderly they were higher (2,010). Delahoy et al. (62) suggested that a reduction of 1 mmol/L in LDL-c was associated with a 21% proportional reduction in the incidence of vascular events. Therefore, based on our present results, it is expected that synbiotic intervention in hypercholesterolemic patients would lead to an approximately 18% reduction in major cardiovascular events if a linear relationship between reduced LDL-c concentrations and reduced incidence of vascular events is assumed.

## Conclusion

We could conclude that *L. rhamnosus* GG combined with SCF intervention could be useful in the primary intervention of hypercholesterolemia and may lead to reductions in risk factors for CVD. The observed reduction of TC and LDL-c by *L. rhamnosus* GG combined with SCF and not *L. rhamnosus* GG-PB12 is of interest and may have various mechanistic explanations. First, *L. rhamnosus* GG showed a much higher mucus-binding capacity than *L. rhamnosus* GG-PB12 (19). Hence, *L. rhamnosus* GG is expected to reside in close proximity to intestinal cells, allowing for increased interactions. Second, the increased persistence of *L. rhamnosus* GG may also impact the extent of host interactions. Both of these explanations imply that *L. rhamnosus* GG has beneficial host signaling capacity that affects TC and LDL-c concentrations. Third, *L. rhamnosus* GG has a differential effect on the total intestinal community and hence it cannot be excluded that the affected microbial community is involved in reduction of hypercholesteremia. Whatever the exact mechanism would be, the observation that TC and LDL-c concentrations were only reduced by an intervention with *L. rhamnosus* GG and not *L. rhamnosus* GG-PB12, provided evidence that the mucus-binding pili may confer a health influence. In conclusion, the synbiotic containing *L. rhamnosus* GG combined with SCF showed a tendency to promote innate immunity by increasing NK cell activity in elderly women and in 70- to 80-year-old volunteers and decreased TC and LDL-c in hypercholesterolemic patients. In addition, *L. rhamnosus* LGG-PB12 combined with SCF demonstrated an increase in NK cell activity compared to SCF alone in older volunteers. We also found significant positive effects on the immune response, evidenced by a decrease of the pro-inflammatory cytokine IL-6. Therefore, dietary intervention with *L. rhamnosus* GG combined with SCF could be of importance in elderly as an attractive option for enhancement of both the microbial and immune systems.

## Ethics Statement

The study was registered as a clinical trial (http://clinicaltrials.gov ID: NCT03168503) and was conducted according to the Declaration of Helsinki following GCP. The study was approved by the University of Reading Research Ethics Committee (Ethics reference number UREC14/06).

## Author Contributions

AC was the principal investigator of the study; AC and GG designed research; TB-M, AC, PR, KK, and WV conducted research, analyzed data, and performed statistical analysis; TB-M, AC, and GG wrote the paper; AC had primary responsibility for the final content. All authors read and approved the final manuscript.

## Conflict of Interest Statement

The funders of this study had no input on the design, implementation, analysis, or interpretation of the data. The authors have no other conflicts of interest to declare.
