# Supplementary material for: Effects of Soluble Corn Fiber Alone or in Synbiotic Combination with Lactobacillus rhamnosus GG and the Pilus-Deficient Derivative GG-PB12 on Fecal Microbiota, Metabolism, and Markers of Immune Function: A Randomized, Double-Blind, Placebo-Controlled, Crossover Study in Healthy Elderly (Saimes Study)
Source: Front Immunol. 2017 Dec 12;8:1443. doi: 10.3389/fimmu.2017.01443 (PMC5733116; doi:10.3389/fimmu.2017.01443)
Supplement: Supplementary file 1 [file data_sheet_1.docx]

**Figure S1.** **Calibration curves of *Lactobacillus rhamnosus* GG and its derivative mutant GG-PB12: qPCR assays used in the study.**

*L. rhamnosus* GG


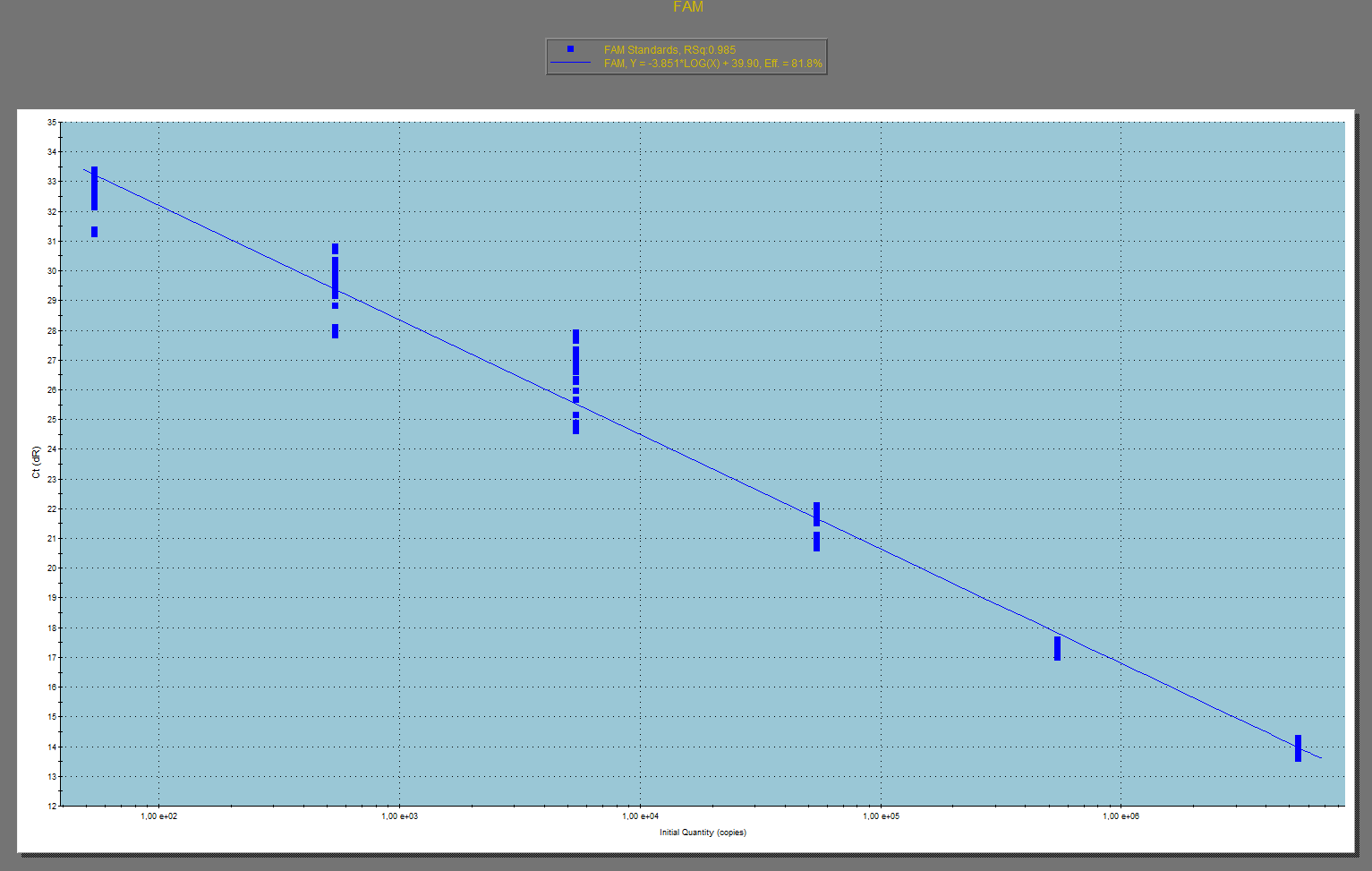


RSq: 0.985

Y= -3.851*LOG(X)

+ 39.90

Eff= 81,8 %

*L. rhamnosus* GG-PB12
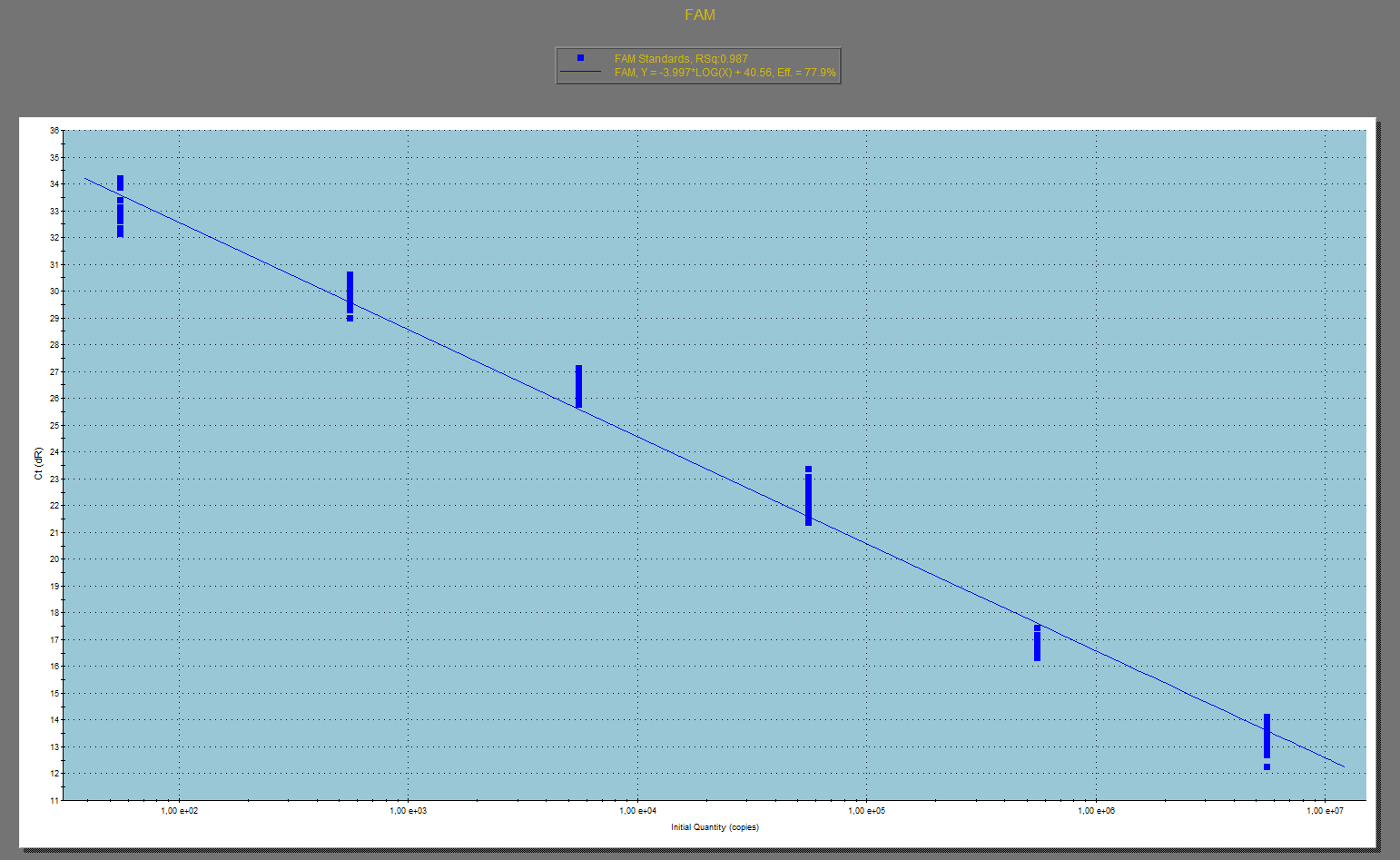


RSq: 0.987

Y= -3.997*LOG(X)

+ 40.56

Eff= 77.9 %
